# Exogenous polyserine and polyleucine are toxic to recipient cells

**DOI:** 10.1038/s41598-022-05720-y

**Published:** 2022-01-31

**Authors:** Ryuji Owada, Shinichi Mitsui, Kazuhiro Nakamura

**Affiliations:** 1 Department of Laboratory Sciences, Gunma University Graduate School of Health Sciences, 3-39-22, Showa-machi, Maebashi, Gunma 371-8511 Japan; 2 Department of Rehabilitation Sciences, Gunma University Graduate School of Health Sciences, 3-39-22, Showa-machi, Maebashi, Gunma 371-8511 Japan

**Keywords:** Neuroscience, Medical research, Neurology, Pathogenesis

## Abstract

Repeat-associated non-AUG (RAN) translation of mRNAs/transcripts responsible for polyglutamine (polyQ) diseases may generate peptides containing different mono amino acid tracts such as polyserine (polyS) and polyleucine (polyL). The propagation of aggregated polyQ from one cell to another is also an intriguing feature of polyQ proteins. However, whether the RAN translation-related polyS and polyL have the ability to propagate remains unclear, and if they do, whether the exogenous polyS and polyL exert toxicity on the recipient cells is also not known yet. In the present study, we found that aggregated polyS and polyL peptides spontaneously enter neuron-like cells and astrocytes in vitro. Aggregated polyS led to the degeneration of the differentiated neuron-like cultured cells. Likewise, the two types of aggregates taken up by astrocytes induced aberrant differentiation and cell death in vitro. Furthermore, injection of each of the two types of aggregates into the ventricles of adult mice resulted in their behavioral changes. The polyS-injected mice showed extensive vacuolar degeneration in the brain. Thus, the RAN translation-related proteins containing polyS and polyL have the potential to propagate and the proteins generated by all polyQ diseases might exert universal toxicity in the recipient cells.

## Introduction

The mutant genes responsible for polyglutamine (polyQ) diseases contain expanded CAG repeats. Several neurodegenerative diseases such as autosomal dominant forms of several spinocerebellar ataxia (SCA), spinal and bulbar muscular atrophy, Huntington’s disease (HD) and dentatorubropallidoluysian atrophy are categorized as polyQ diseases^1^. The severity and time of onset of the symptoms of polyQ diseases are in proportion to the repeat number^2^ and the pathogenic repeat number of glutamine is over 36 in the case of HD^1^. The pathogenesis of polyQ diseases has been extensively investigated^1,3–14^ and the expanded polyQ-containing proteins are known to aggregate in neurons and exert cytotoxicity. In addition, mutant Huntingtin (HTT), the causative molecule of HD, promotes autonomous microglia activation^2^. The toxicity elicited by CAG repeat expansions has been believed to be solely ascribed to translation to create polyQ proteins. However, recent studies have clarified that proteins with homopolymeric sequences other than polyQ are also translated from the CAG repeat-containing genes by repeat-associated non-AUG (RAN) translation^15^. RAN translation is also seen in the causative gene for other neurodegenerative disorders such as amyotrophic lateral sclerosis and frontotemporal dementia^16^. In case of RAN translation occurring at sense and antisense transcripts of mutant genes responsible for polyQ diseases, there may be theoretically generated peptides containing homopolymeric polyQ, polyleucine (polyL), polyserine (polyS), polycystein (polyC) and polyalanine (polyA) tracts. Upon the RAN translation, the sense transcripts generate polyQ, polyS and polyA, and the antisense transcripts generate polyL, polyC and polyA^17^. Indeed, the four different homopolymeric peptides generated as a result of RAN translation of HTT mutant transcripts were reported to accumulate in the caudate putamen, white matter and the cerebellum in the brain of HD patients^17^. Importantly, neuronal loss, apoptosis and microglial activation were observed in these brain regions, and the individual RAN protein with a C-terminal region was found to be toxic to neurons^17^. Likewise, RAN proteins with polyQ and polyA were reported to be generated from the ataxin 3 gene, a gene responsible for spinocerebellar ataxia (SCA) type 3, and the RAN proteins induced apoptosis^18^. These observations indicate that endogenous RAN proteins are toxic.

Interestingly, aggregated HTT with expanded polyQ has the ability to propagate between cells^19^. Transfer between cells has also been reported in other misfolded proteins that are responsible for neurodegenerative disorders^20^. However, whether this is also the case for RAN proteins generated from genes for polyQ diseases has not been determined yet. It is also unclear whether exogenous RAN proteins are toxic to recipient cells. Since toxicity is shared by different RAN proteins with different C-terminal sequences, it is likely that the homopolymeric sequences themselves without the flanking sequences in RAN proteins are determinants of toxicity to cells. Homopolymeric peptides are useful model molecules to address this assumption. Moreover, the strategy of using the peptides also simplifies the experimental setup.

In the present study, we prepared polyS and polyL peptides as products of RAN translation from polyQ disease-related genes, and the peptides were aggregated. Then, electron microscopic analysis revealed the entry of the two types of aggregates into neuron-like cells and astrocytes. Functional analyses such as cell death, degeneration and differentiation were performed using the recipient cells. Finally, we introduced the two types of aggregates into the ventricles of adult mice and conducted behavioral analyses such as tests for anxiety and depression.

## Results

### 13S and 13L display distinct internal structure of aggregates

**Figure 1:**
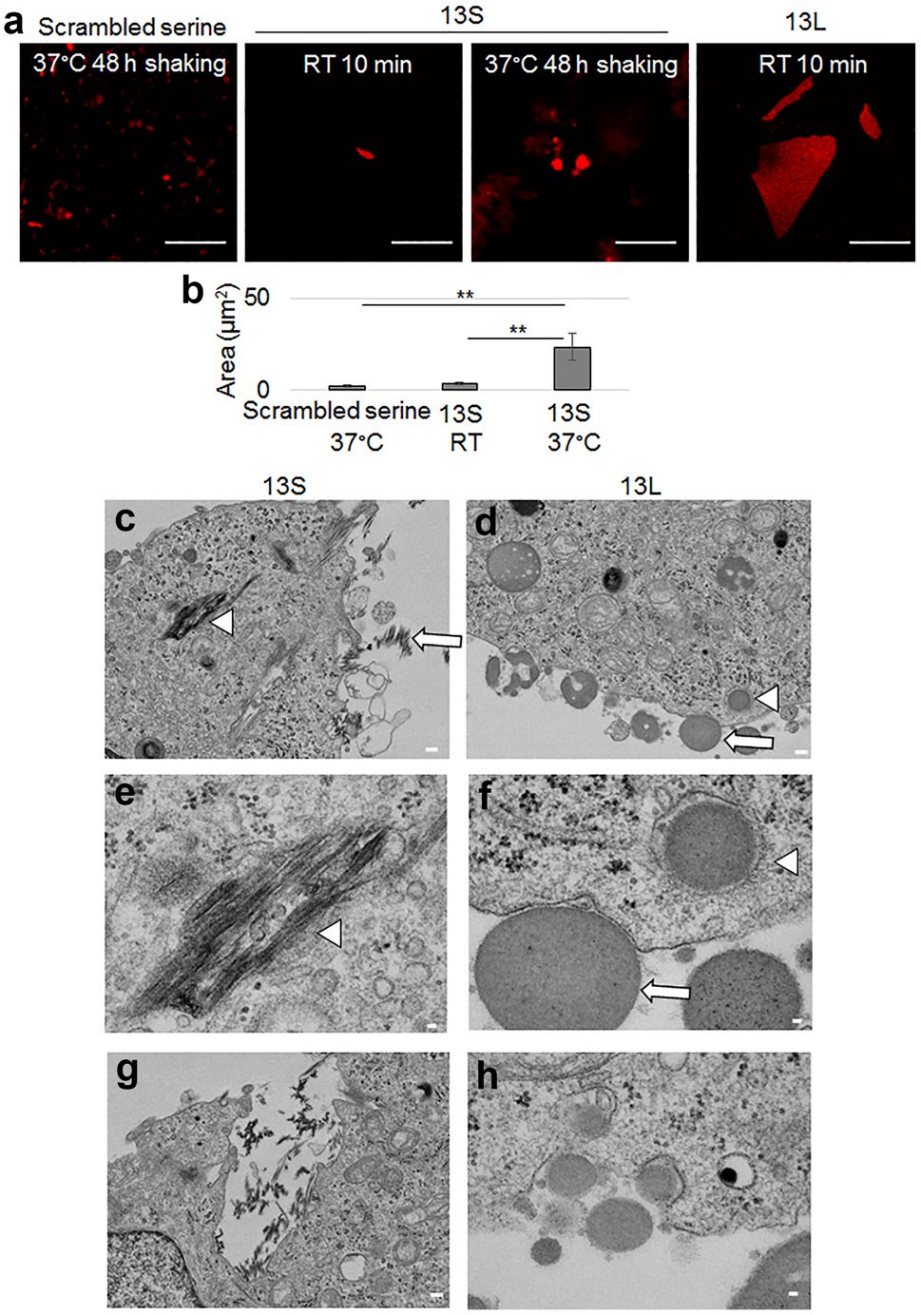
Aggregated 13S and 13L enter PC12 cells. (**a**,**b**) Fluorescent images of clusters of aggregates of TAMRA-labeled 13S and 13L peptides (1 mg/ml in water, red). 13S and 13L were incubated for 10 min at room temperature. 13S and scrambled serine were also incubated for 48 h at 37 °C with shaking (**a**). Area of aggregates were quantified (n = 20 each) (**b**). ANOVA, **p < 0.01. Scale bar, 20 µm. (**c**–**h**) Transmission electron microscopic images of undifferentiated PC12 cells having 13S or 13L. 13S peptide (1 mg/ml in water) was incubated for 48 h at 37 °C with shaking. Then, the aggregated 13S peptide was added in the culture medium of PC12 cells to be at a final concentration of 10 µg/ml. The stock solution of 13L peptide was directly applied in the culture medium to be at a final concentration of 10 µg/ml. The cells having two peptides were incubated for 1 day at 37 °C. Extracellular aggregates (arrows) of 13S (**c**) and 13L (**d**) are shown. 13S (**c**,**e**,**g**) and 13L (**d**,**f**,**h**) aggregates inside the cells (arrowheads) are also shown. The invaginated vesicles having aggregates inside are also shown (**g**, 13S; **h**, 13L). Scale bars, 20 µm (**a**), 1 µm (**c**,**d**,**g**), 200 nm (**e**,**f**,**h**).

The amino acid repeats need to be aggregated to allow the RAN translation-generated amino acid repeats to enter the cells. Among RAN translation-generated amino acid repeats from CAG repeat-containing genes, we focused on polyL because polyL encoded by mixed DNA repeats displayed higher toxicity than polyQ in mammalian cells^21^. Because we aimed to search for common toxicity of polyL-containing proteins to cells, we decided to use a short peptide containing a leucine repeat without the following C-terminal long sequence. Although a synthetic polyQ peptide with 15 glutamine repeats exhibited weak aggregation^23^, even a short repeat of leucine was, however, expected to form strong aggregates on account of its hydrophobic property. A previous study showed that a peptide with 13 alanine repeats (KKWA_13_KK) formed solid aggregates^22^. Therefore, we followed the flanking short sequences (KKW and KK) and a repeat number of 13 for the polyL peptide (KKWL_13_KK). In addition, the polyL (13L) was labeled with the fluorescent dye TAMRA to enable detection of polyL aggregates by fluorescence microscopy. Based on the observation that a polyQ peptide with 69 glutamine repeats formed aggregates in aqueous solution^23,24^, we initially tested the formation of 13L aggregates in water at a concentration of 1 mg/ml. Generally, the degree of aggregation becomes stronger with long incubation times, high temperatures and shaking. As shown in Fig. 1a,b, even incubation of 13L in water for 10 min at room temperature without shaking generated large clusters of aggregates such as spherical agglomerates. The largest area of the cluster of 13L was approximately 500 µm^2^. Since such clusters are not able to enter cultured cells, we decided not to induce aggregation of 13L at 1 mg/ml before application to cultured cells. Instead, the stock solution of 13L peptide was directly applied to the culture medium to obtain a final concentration of 10 µg/ml for the subsequent functional analyses using cultured cells. As a reference, we prepared the 13S peptide (KKWS_13_KK) and induced aggregation under the same conditions (10 min at room temperature). However, this condition generated much smaller clusters of aggregates than those of 13L (Fig. 1a,b). Therefore, we applied the another condition for the 13S. When 13S was incubated for 48 h at 37 °C with shaking, a rod-like larger cluster of aggregates was detected (Fig. 1a,b). A scrambled serine peptide (SSSKSSKSKSSSWSSSKS) with the same set of amino acids as 13S was also prepared as a control for 13S and compared the degree of aggregation between 13S and the scrambled serine under incubation at 37 °C for 48 h with shaking. Although even the scrambled serine formed small cluster of aggregates, the area of the cluster of 13S was significantly larger than the scrambled serine (Fig. 1a,b). Thus, the 13S and scrambled serine (1 mg/ml) were induced to aggregate under the conditions above for the subsequent functional analyses using cultured cells.

### Internalized 13S leads to retraction of neurites of differentiated PC12 cells

PolyQ diseases manifest as age-related and progressive degeneration of neurons. We addressed whether RAN translation-related 13S and 13L also led to cell degeneration. To induce internalization of 13S and 13L, these peptides were added to the culture medium of undifferentiated PC12 cells and were cultured for 1 day. Transmission electron microscopy images verified that the appearance of 13S aggregates outside PC12 cells resembled fibrillar aggregates (Fig. 1c). Likewise, the internal structure of the 13L aggregates was found to be complex with many grains and fibers inside (Fig. 1d,f). Again, the two peptides showed different internal structures.

Remarkably, the 13S (Fig. 1c,e) and 13L (Fig. 1d,f) aggregates found in the cytoplasm of PC12 cells had identical electron densities and internal structures as those present outside the cells. These results indicated that the two types of aggregates were definitely taken up by undifferentiated PC12 cells. Notably, the aggregates were often found in the closed bilayer vesicle-like structures in the cytoplasm. Occasionally, the aggregates were also found in the invaginated pits (Fig. 1g,h).

**Figure 2:**
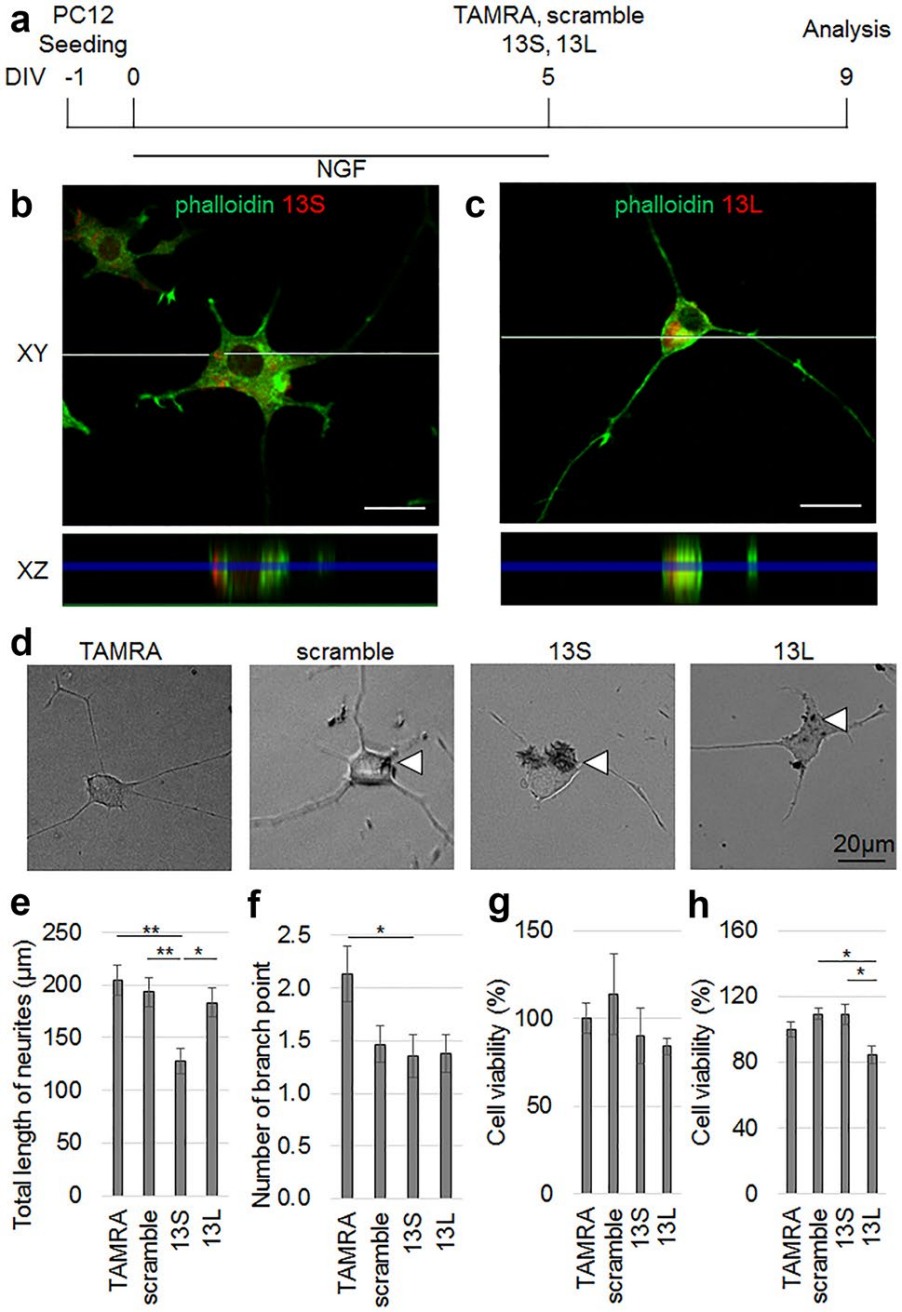
Entry of aggregated 13S leads to retraction of neurites of differentiated PC12 cells. (**a**) Time schedule of the experiment. (**b**, **c**) z-stacks confocal images of differentiated PC12 cells with an interval of every 1 µm were obtained on day 4 after application with the peptides. Ortho XZ-axis cross section images (bottom) along the white line in the XY-axis view (top) were reconstituted from the z-stacks. The 13S (**b**) and 13L (**c**) signals (red) are located between phalloidin signals (green). (**d**–**f**) Morphology of differentiated PC12 cells given TAMRA, scrambled serine, 13S or 13L (n = 45 cells, each from 3 experiments). Representative images are shown in (**d**). The morphological indices that reflect degeneration of neurons are total length of neurites (**e**) and number of branch point (**f**). Arrowheads indicate aggregates. (**g**,**h**) The viability of differentiated (n = 5, 10, 5 and 5 for TAMRA, scrambled serine, 13S and 13L, respectively) (**g**) and undifferentiated (n = 5 each) (**h**) PC12 cells were studied at 1 day after application of the peptides. The values relative to those of TAMRA-treated cells are expressed. Error bars represent SE. ANOVA, *p < 0.05, **p < 0.01. Scale bars, 20 µm.

To mimic the neural degeneration in vitro, we tested the retraction of neurites. We fully differentiated PC12 cells to avoid extending neurites, and then added 13S or 13L. PC12 cells were cultured for 5 days in the presence of NGF. Then, aggregated 13S and 13L were added to the culture medium. The cells were further cultured for 4 days without NGF (Fig. 2a). To confirm that 13S and 13L aggregates were incorporated into differentiated PC12 cells, the orhto images along the vertical axis were constructed from z-stack confocal images taken at every 1 µm (Fig. 2b,c for 13S and 13L, respectively). We used phalloidin, an actin-binding reagent, as a marker for the cytoplasm. The fluorescent signals of the two types of aggregates were located between phalloidin signals, suggesting that 13S and 13L aggregates are located inside differentiated PC12 cells. The percentages of cells that incorporated 13S and 13L were 98% and 100%, respectively.

As part of the functional analyses of the differentiated PC12 cells with 13S or 13L, retraction of neurites was estimated by the total length of neurites of the differentiated PC12 cells. Since the scrambled serine was also taken up by the cells, we could compare the morphology of the cells with 13S to those with scrambled serine. The length of PC12 cells treated with 13S but not 13L aggregates was significantly shorter than that of TAMRA-and scrambled serine-treated cells (Fig. 2d,e). The number of branch point of neurites in 13S-treated cells were significantly fewer than TAMRA-treated cells (Fig. 2d,f), however the number was not different from that of scrambled serine-treated cells (Fig. 2d,f). The number of 13L-treated cells was not different from any groups (Fig. 2d,f). Thus, it can be concluded that exogenous 13S enters PC12 cells, where the aggregates lead to the retraction of neurites. When the cell viability assay was conducted using a formazan dye generated by the activities of dehydrogenases in cells, neither 13S nor 13L induced significantly lower viability than TAMRA alone and scrambled serine in differentiated PC12 cells (Fig. 2g). In undifferentiated PC12 cells, 13L induced significantly lower viability than scrambled serine. However, the viability was not different from that of TAMRA (Fig. 2h). The morphological difference between the cells with 13S and those with 13L might be due to differences in the internal structure of the two types of aggregates, as evidenced by electron microscopic analysis.

### Incorporation of aggregated 13S and 13L affects differentiation and viability of KT-5 astrocytic cells

**Figure 3:**
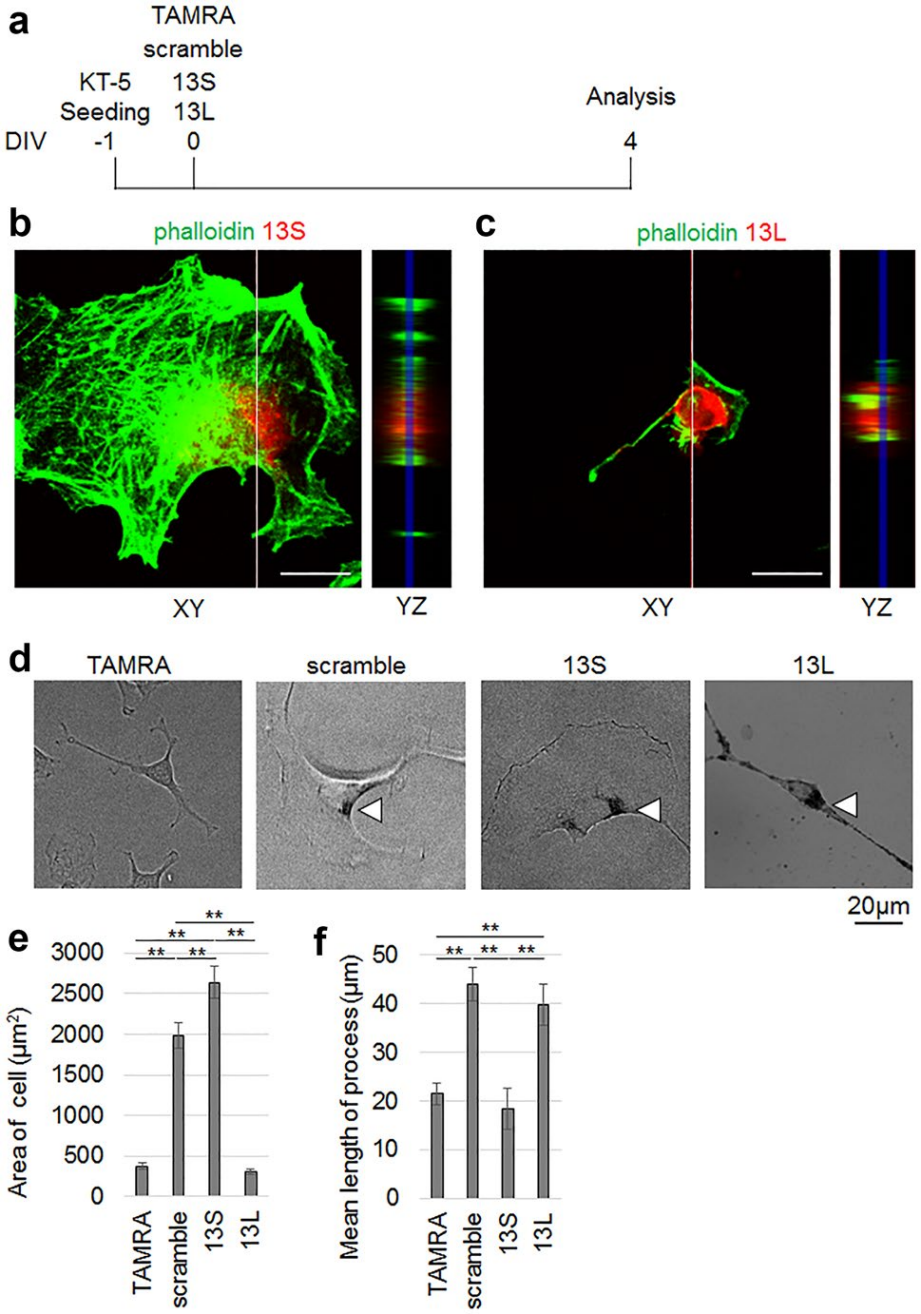
Incorporation of aggregated 13S and 13L affects differentiation of KT-5 astrocytic cells. (**a**) Time schedule of the experiment. (**b**,**c**) Ortho YZ-axis cross section images (right) along the white line in the XY-axis view (left) was reconstructed from the z-stacks. The fluorescent signals of 13S (**b**) (red) and 13L (**c**) (red) are found between phalloidin signals (green). (**d**–**f**) Total area of cell (**e**) and averaged length of each process per cell (**f**) of KT-5 cells given with TAMRA, scrambled serine, 13S or 13L were quantified (n = 30 cells, each from 3 experiments). Representative images are shown in (**d**). Arrowheads indicate aggregates. Error bars represent SE. ANOVA, **p < 0.01. Scale bars, 20 µm.

A previous report demonstrated that polyQ-containing aggregates were found in astrocytes in the brains of patients with HD^25^. Thus, we addressed the question whether RAN translation-related 13S and 13L were taken up by astrocytes and, whether they were also toxic to astrocytes. After addition of aggregated 13S, aggregated 13L, scrambled serine or TAMRA alone to KT-5 astrocytic cells, the cells were further cultured for 4 days (Fig. 3a). Again, ortho-vertical images verified that both 13S (Fig. 3b) and 13L (Fig. 3c) were found between phalloidin signals, indicating that 13S and 13L were taken up by KT-5 cells.

Normal astrocytes show a lattice-like shape. After injuries, the astrocytes are converted into hypertrophic reactive astrocytes^26^. To study parameters that reflected the morphology of the hypertrophic reactive astrocytes, we quantified the total area of cell and average length of each process per cell of KT-5 cells treated with TAMRA, scrambled serine, 13S or 13L. Remarkably, 13S induced marked hypertrophy of the cells, as demonstrated by the larger area of cells than TAMRA alone- and scrambled serine-treated cells (Fig. 3d,e). Even scrambled serine-treated cells showed significantly larger area of the cells than TAMRA-treated cells (Fig. 3d,e). The area was not different in 13L-treated cells compared to TAMRA-treated cells (Fig. 3d,e). However, 13L led to longer processes than TAMRA alone (Fig. 3d,f). Scrambled serine also led to longer processes than TAMRA (Fig. 3d,f).

**Figure 4:**
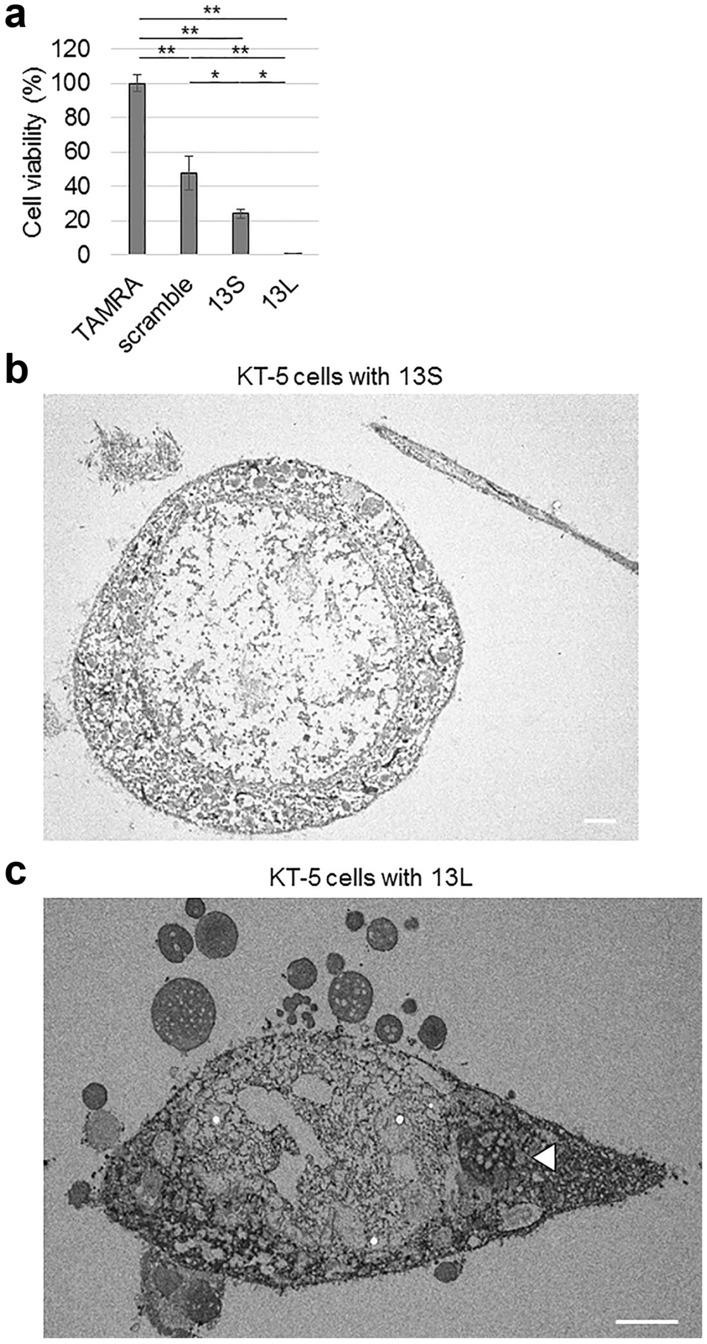
Aggregated 13S and 13L lower viability of KT-5 astrocytic cells. (**a**) Viability of KT-5 cells treated with TAMRA, scrambled serine, 13S or 13L (n = 5, each) was tested 4 days after application of peptides. The values relative to that of TAMRA-treated cells are expressed. Error bars represent SE. ANOVA, *p < 0.05, **p < 0.01. (**b**,**c**) Extensive KT-5 cell death by aggregated 13S (**b**) and 13L (**c**), as revealed by transmission electron microscopy. The peptides were added in the culture medium of KT-5 cells at a final concentration of 10 µg/ml and were incubated for 1 day at 37 °C. An arrowhead indicate aggregates inside KT-5 cells. Scale bars, 5 µm.

We then examined the viability of KT-5 cells treated with TAMRA, scrambled serine, 13S or 13L. Marked lower viability was detected in KT-5 cells with 13L compared to that in those treated with TAMRA alone (Fig. 4a). 13S also led to lower viability than TAMRA alone and scrambled serine (Fig. 4a). Even scrambled serine led to decreased viability than TAMRA (Fig. 4a). Moreover, the extensive cell death by aggregated 13S and 13L was revealed by analysis with transmission electron microscopy. Normal structures of intracellular organelles in the cells with 13S (Fig. 4b) and 13L (Fig. 4c) were completely destroyed, probably due to the breakdown of the membranes of the organelles. These results suggest that the two types of aggregates promote differentiation and cell death in astrocytes.

### Extensive vacuolar degeneration by exogenous 13S in mice

Given the toxicity of 13S and 13L in vitro, the exogenous aggregates might alter the brain functions of adult mice. We first checked the distribution of 13S and 13L injected into the lateral ventricle. We chose the lateral ventricle as the injection site because the lateral ventricle occupies a wide area of the brain; therefore, the aggregates are predicted to spread broadly. The 13S and scrambled serine were induced to aggregate by incubation for 48 h at 37 °C with shaking at 207 rpm (1 mg/ml) and then diluted to 100 µg/ml with PBS for the injection. For 13L, the stock solution of 13L peptide was directly diluted to 100 µg/ml with PBS for the injection.

**Figure 5:**
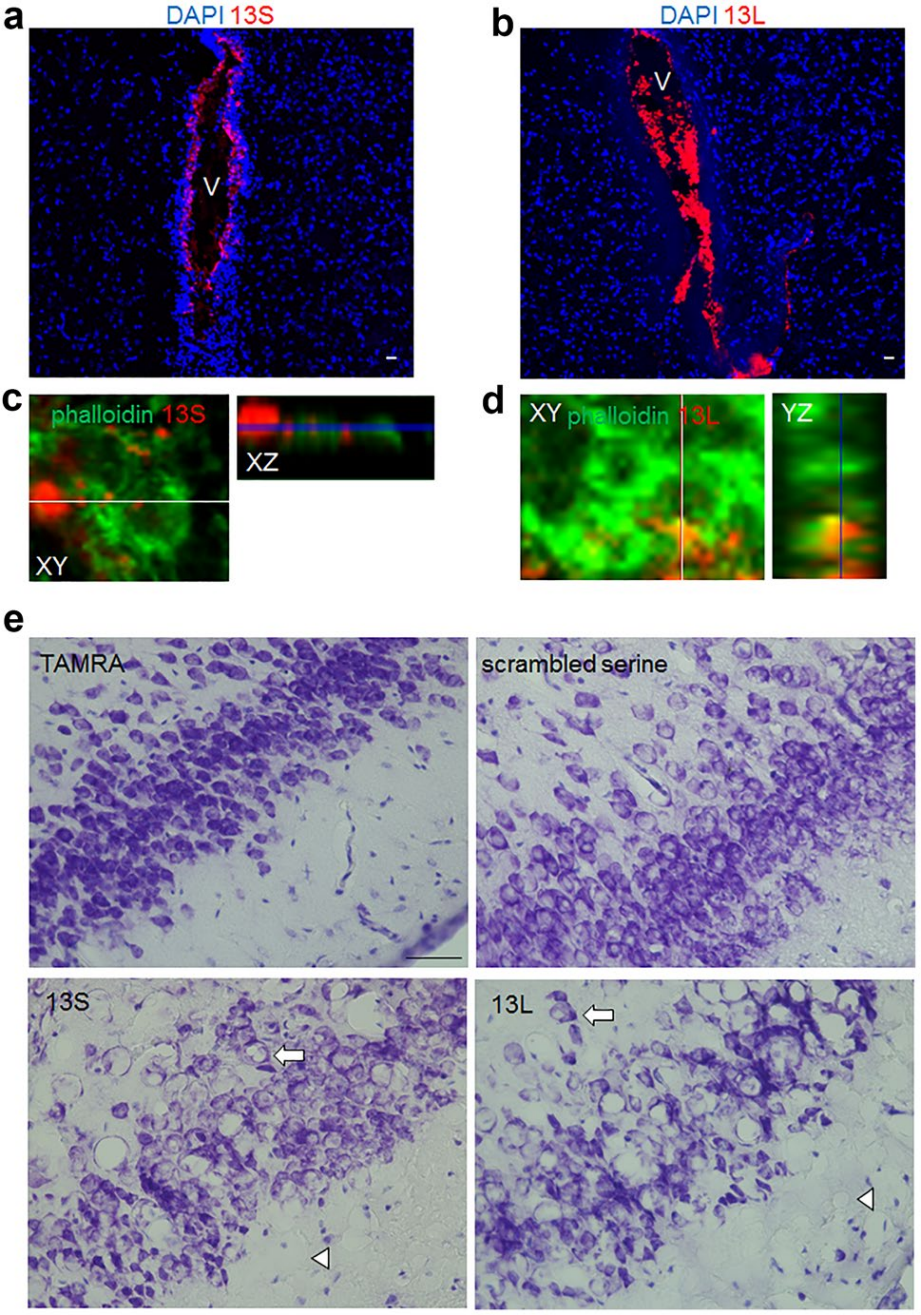
Vacuolar degeneration of the brain given 13S and 13L aggregates. (**a**–**d**) Fluorescence images of coronal brain sections of mice to which aggregated 13S (red) (**a**,**c**) or 13L (red) (**b**,**d**) was injected into the lateral ventricle 1 day before. The sections were stained with phalloidin (green) and DAPI (blue). The 13S (**a**) and 13L (**b**) signals are found in the lateral ventricle (V). Ortho XZ or YZ-axis cross section confocal images along the white line in the XY-axis view proved localization of 13S (**c**) and 13L (**d**) in the phalloidin-positive cytoplasm. (**e**) Nissl-stained coronal sections of mice treated with TAMRA, scrambled serine, 13S or 13L. Images were taken from the piriform cortex. The arrows and arrowheads indicate vacuoles inside and outside the soma of neurons, respectively. Scale bars, 20 µm (**a**,**b**), 50 µm (**e**).

TAMRA-conjugated 13S and 13L were found in the lateral ventricle the next day (Fig. 5a,b). Aggregates were detected in the brain parenchyma close to the ventricle. The confocal ortho image clarified the localization of 13S and 13L between actin-binding phalloidin signals (Fig. 5c,d), indicating that the aggregates were incorporated into cells.

We then performed Nissl staining to check if 13S and 13L led to changes in neuronal architecture. Notably, many vacuoles were widely found in the Nissl-stained cerebrum in 13S-injected mice 1 day after injection (supplementary Fig. S1a online). The vacuoles were also observed in scrambled serine-injected brain, but to a lesser extent than 13S (supplementary Fig. S1a online). Magnified images of Nissl-stained sections verified that the vacuoles were located in the cell body of neurons in the motor cortex (supplementary Fig. S1b online), hippocampus (supplementary Fig. S1c online) and striatum (supplementary Fig. S1d online) of 13S-treated mice, suggesting vacuolar degeneration of neurons. There were also large vacuoles outside the neuronal soma (supplementary Fig. S1d online). Some vacuoles might contain cytoplasm or proteins because the vacuoles were eosin-positive in HE-stained sections (supplementary Fig. S1e online). The vacuoles in the neuronal soma were detected in the piriform cortex of 13L-injected brain 1 day after injection (Fig. 5e), although the vacuoles were rarely detected in other regions (supplementary Fig. S1a online). The vacuolar degeneration was also seen in the piriform cortex from scrambled serine-injected mice, but to a lesser extent than 13S (Fig. 5e).

### Altered behavior by exogenous 13S and 13L in mice

**Figure 6:**
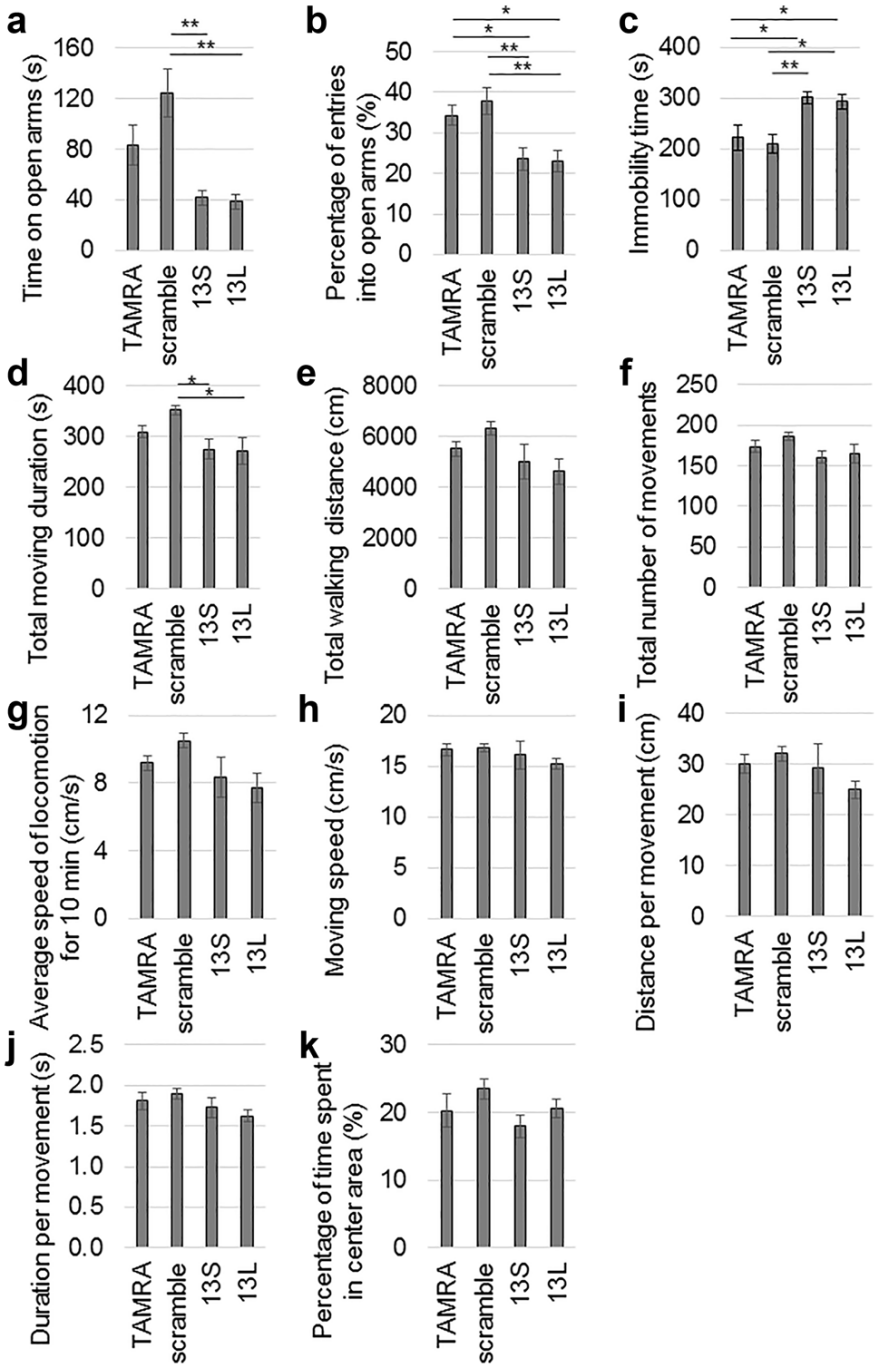
Altered performances of elevated plus maze and forced swimming tests by 13S and 13L. (**a**,**b**) Elevated plus maze test. Time on open arms (**a**) and percentage of the number of entries into the open arms out of the number of entries into all 4 arms (**b**) were measured in mice given TAMRA, scrambled serine, 13S or 13L (n = 18 each). (**c**) Immobility time during forced swim test were measured in mice given TAMRA, scrambled serine, 13S or 13L (n = 18 each). (**d**–**k**) Open field test. Spontaneous motor activity of the mice were evaluated in an open field for 10 min. The total moving duration (**d**), total walking distance (**e**), total number of movements (**f**), average speed of locomotion for 10 min (**g**), moving speed (**h**), distance per movement (**i**), duration per movement (**j**) and percentage of time spent in the center area (**k**) were measured in mice in which TAMRA (n = 9), scrambled serine (n = 10), 13S (n = 10) or 13L (n = 10) was injected. Error bars represent SE. ANOVA, *p < 0.05, **p < 0.01.

Since significant vacuolar degeneration was extensively detected the next day after the injection of 13S, we decided to carry out behavioral tests the next day. Multiple regions including the piriform cortex are involved in emotion. Therefore, we tested anxiety, depression and stress-coping behavior. For elevated plus maze test, the mouse was first placed in the center area and was allowed to enter the open and closed arms. If the mouse had increased anxiety, the time spent on the open arms became shorter. Both 13S-injected and 13L-injected mice spent a shorter time spent on the open arms than scrambled serine-injected mice (Fig. 6a). In addition, the percentage of the number of entries into the open arms out of the number of entries into all four arms also decreased in both 13S-injected and 13L-injected mice than TAMRA- and scrambled serine-injected mice (Fig. 6b). These results suggest an increase in anxiety. We next employed the forced swim test, a test for depression^27^ or a stress-coping strategy^28^. Both 13S-injected and 13L-injected mice spent longer immobility time in the water than TAMRA- and scrambled serine-injected mice (Fig. 6c), indicating that the injected mice were depressed or they applied different stress-coping strategies in the water.

The vacuoles were found in the striatum, motor cortex and hippocampus in 13S-treated mice. These regions control and regulate motor activity. Therefore, we checked the general motor activity using an open-field test. Although total moving duration was significantly lower in 13S- and 13L-treated mice than scrambled serine-treated mice, these values were not different from those of TAMRA (Fig. 6d). 13S- and 13L-injected mice did not show differences in total walking distance (Fig. 6e), total number of movements (Fig. 6f), average speed of locomotion for 10 min (Fig. 6g), moving speed (Fig. 6h), distance per movement (Fig. 6i) and duration per movement (Fig. 6j) compared with TAMRA-injected mice. Therefore, we can exclude a possibility that the change in spontaneous activity in 13S- and 13L-treated mice might affect behavioral results seen in elevated plus maze and forced swim tests. We then checked the percentage of time that mice stayed in the center area because mice with enhanced anxiety tended to dislike the center area in the open field. However, there were no significant differences among the four groups (Fig. 6k). Thus, enhanced anxiety of 13S- and 13L-injected mice seems not to be generally observed but to be seen in specific fearful situations.

## Discussion

The existence of RAN proteins from the genes for polyQ diseases has been proven using cell models^18^, animal models and human tissues. Animal studies have included SCA8 and myotonic dystrophy type 1 (DM1)^29^. Accumulation of SCA8 polyA and DM1 polyQ have been observed in previously established mouse models^29^. Endogenous RAN proteins have been reported to exert toxicity in various models^17,18^. In contrast, we explored the potential toxicity of exogenous RAN proteins in recipient cells.

Cell-to-cell transmission is a pivotal phenomenon shared by aggregated proteins that are causative for neurodegenerative disorders including polyQ diseases^19^. PolyQ-expanded Htt is reported to be released from presynaptic terminals and it is taken up by phagocytic glia in *Drosophila*^30^. We showed evidence of the uptake of peptides that are related to RAN translation of CAG repeat-containing genes by astrocytic glial cells. In the *Drosophila* model^30^, the authors revealed that the phagocytic glia could regulate the amount of Htt aggregates located in the neurons. Intriguingly, the clearance by glia is dependent on Draper, the scavenger receptor, and the downstream phagocytic engulfment machinery^30^. Since we added the 13S and 13L to the culture medium and observed the entry of these aggregates into PC12 cells and astrocytes, it remained an open question as to whether these aggregates were also taken up by astrocytes in animal brains.

The precise mechanisms by which the polyS and polyL pass through the cell membrane bilayer of neurons and astrocytes are elusive. The interaction between polyQ aggregates and proteins on cell membrane is suggested to contribute to the efficient internalization^19^. As a mechanism to bring the aggregates to the cytoplasm after the interaction, exogenous HTT Exon1 aggregates were found to be taken up by clathrin-dependent endocytosis, and they were eventually transferred to lysosomal compartments in cultured cells^31^. Clathrin-mediated endocytosis requires dynamin to form a ring on the neck of the invaginated pits. The vesicles are then pinched off from the plasma membrane^32^. Electron microscopic analysis of PC12 cells revealed that 13S and 13L aggregates were located in closed vesicle-like structures. The membrane of the vesicle resembled a cell membrane because of its bilayer structure. Moreover, we also detected the pits that were invaginated from the cell membrane and which contained the aggregates inside them. Thus, the entry of the polyS and polyL aggregates in PC12 cells is likely endocytosis. We have not determined whether the endocytosis is dependent on clathrin or caveolin. There is a need for determination of the complex modular protein machinery required for clathrin-coated endocytic vesicles^33^ upon application of the aggregates to PC12 cells. However, whether the entry in KT-5 astrocytes used endocytosis has not been determined because the vast majority of KT-5 cells with the aggregates were dead, and we could not detect intact vesicle-like structures.

In the phagocytic glial cytoplasm of the *Drosophila* model of HD^30^, the engulfed exogenous Htt aggregates caused prion-like conversion of soluble, normal Htt. The prion-like conversion has a pathological significance because this phenomenon might lead to the spread of initial focus of polyQ aggregates in the brain. It is interesting to examine the prion-like conversion and release from cells using polyS and polyL.

When RAN translation initiation of ataxin 3 was examined in various cellular models expressing non-canonically translated sequences of ataxin 3, the sequences surrounding the repeat region were demonstrated to have an impact on the translation initiation site and efficiency. Activation of the integrated stress response enhanced the RAN translation^18^. Moreover, although RAN translation with polyQ repeats started at non-cognate codons located upstream of the CAG repeats, RAN polyA proteins were likely translated within the repeats^18^. Therefore, we used the polyS and polyL peptides with only three amino acids before the repeats. However, it should be noted that when the RAN products are endogenously generated in polyQ diseases, the products may contain flanking specific sequences. In addition, when mutant CAG tracts in polyQ-causative proteins are RAN-translated, repeat numbers of the products are likely longer than 13. These points are limitations of the current simple procedure for mimicking cells with the RAN products. Nevertheless, homopolymeric polyS and polyL are useful for analyzing the universal toxicity of the RAN products from all polyQ diseases. We chose to use polyS and polyL peptides, although RAN translation from the causative genes for polyQ diseases also creates polyA and polyC. Aggregation of polyA with 37 alanine repeat was reported to be mechanistically dissimilar to that of polyQ^34^. Nevertheless, impaired neuron-like differentiation of PC12 cells with polyA was also detected^35^. As for polyC, a peptide with 13 cysteine repeats and the same flanking sequences as polyS and polyL, KKWCCCCCCCCCCCCCKK, could not be obtained. Since the synthesis of peptides containing cysteine repeats seems to present special challenges, the toxicity of exogenous polyC to the cells might not be tested. However, the toxicity of endogenous polyC using a gene expression system could be investigated.

The 13S and 13L peptides, especially the 13L peptide, were prone to aggregation. This might be owing to the beta-sheet structure of the peptides because analysis of the domain containing seven tandem leucine-rich repeats of internalin B from the bacterium *Lysteria monocytogenes* revealed that each repeat contributed to a single beta-strand that formed a continuous beta sheet with neighboring repeats^36^. Similarly, NMR and X-ray studies provided a structural model in which phosphorylated serine repeats (pSX)4 complex with divalent cations Ca^2+^ and Mg^2+^ to form rigid nanocrystalline beta sheet structures in caddisfly silk^37^. Structural studies using polyS and polyL peptides will be required to clarify this point in the future.

Aggregate formation likely contributes to the pathogenesis of RAN proteins because both aggregate formation and toxicity to cells of individual RAN-associated HTT proteins have been reported in literature^17^. In line with this observation, the 13S and 13L peptides we used in this study were aggregated before their introduction to cells and when the aggregates were taken up by the recipient cells, they exerted toxicity to multiple types of cells. In contrast, it was reported that only polyL was consistently toxic across multiple types of cells, although all RAN polypeptides successfully formed aggregates in the *C. elegans* model^38^. Therefore, the formation of aggregates in endogenous RAN proteins does not seem to be the sole determinant of toxicity. In the case of exogenous RAN proteins, aggregate formation is likely required for the internalization. The type of aggregates of exogenous RAN proteins might confer different degrees of toxicity to the recipient cells.

Neurodegeneration leads to neuronal dysfunction and cell death. 13S induced retraction of neurites of PC12 cells, as seen in exogenous polyQ with 69 glutamine repeats^23^. In line with the retraction of neurites, massive vacuolar degeneration of neurons was found in 13S-injected mice one day after the injection. In addition to the small vacuoles in the neuronal soma, large vacuoles were also found outside the cell body in 13S-treated mice. We have not determined whether the large vacuoles are status spongiosus in which irregular cavities are surrounded by a meshwork of glia^39^ or are the product of cell loss. Future detailed studies using electron micrograph will clarify this point.

Generally, polyQ diseases are age-dependent and therefore, morphological changes are seen in the aged patients and the genetically-engineered animals. Age-dependency is mainly due to the requirement of a long time for endogenous polyQ to form aggregates in the brain. However, because we had injected already aggregated peptides into the brain, even a short period after the injection might be enough to induce detrimental effects. Our experimental design only observed the acute effects of the aggregates on the brain. The chronic effects of the aggregates may be different from the acute effects. Since injection of 13S was only once, appearance of vacuoles might be transient and disappear at later time point.

In contrast to the marked effects of 13S for PC12 cells, the neurite retraction was not caused by application of 13L in PC12 cells. The vacuoles in 13L-injected mice were seen in the piriform cortex but were rarely seen in other regions such as the striatum one day after the injection. Both in vitro and in vivo studies were done a couple of days after application of 13L. A possibility remains that the 13L aggregates might exert toxicity at later time points.

Formation of aggregates is likely the mechanism by which 13S induces vacuolar degeneration of neurons. Vacuolar degeneration is commonly seen in prion diseases and also in neurodegenerative disorders^39^. These disorders are associated with protein aggregates. Intracellular accumulation of disease-associated form of prion protein lead to cytopathology^40^. Because 13S aggregates were found to enter some cells, the aggregates inside the cells might be a direct cytotoxic factor. However, the vacuoles were found in the regions that are distant from the injection site of 13S. Thus, we cannot exclude a possibility that besides incorporated 13S, non-aggregated 13S located in extracellular space and those in the cerebrospinal fluid might contribute to the cytotoxicity. Consistently, tissue damage in prion disease may result from several parallel, interacting or subsequent pathways^40^.

Neurodegeneration triggers universal glial reactions called astrogliosis, by which glial cells affect the progression and outcome of neuropathology^40^. Since astrogliosis is a spectrum of heterogeneous changes, the cells show various appearances^41^. The 13S induced marked hypertrophy of the cell, whereas 13L led to longer processes. Thus, these two types of aggregates likely have different effects on astrocytes. We have not determined whether the changed astrocytes have beneficial or harmful effects on the neighboring cells. Notably, astrocytes expressing mutant Htt in mice became reactive astrocytes that were characterized by a larger size of soma^42^, as seen in 13S-treated astrocytes. Because transport of glutamate was impaired in these mice^42^, the 13S-treated astrocytes might have harmful effects on neighboring neurons. The scrambled serine having multiple four consecutive serine also exhibited morphological changes of KT-5 cells. This observation is not surprising because the peptide can aggregate and enter the cells. Although hypertrophy of the cell was shared by scrambled serine and 13S, the effect was bigger in 13S than scrambled serine. In contrast, longer process was observed in scrambled serine but not in 13S. Thus, the aggregates consisting of different length of serine exert distinct effects on astrocytes.

Furthermore, we found that exogenous 13S and 13L induced severe cell death in KT-5 cells. To date, many types of cell death have been proposed in literature. Cell death can be divided into apoptosis and accidental cell death^43^. Based on the classification of electron microscopy images, cell death can be divided into four types; oncosis (necrosis), typical apoptosis, apoptosis with secondary degeneration, and severely degenerated cells in which differentiation between oncosis and apoptosis is impossible^43^. Although we cannot clearly define the type of the cell death of KT-5 cells, the dead KT-5 cells in electron microscopic images seem to be severely degenerated cells. In contrast, cell death was not observed in PC12 cells treated with either 13S or 13L at least within a couple of days. However, cell death might be induced at later time points because vacuolar degeneration accompanies neural and/or glial cell death in spongiform encephalopathy^39^.

We found behavioral changes in tests for emotion. Higher anxiety in the 13S- and 13L-injected mice were seen in elevated plus maze test that is used to evaluate anxiolytic agents^44,45^. A longer immobility time was also observed in the forced swim test in mice with 13S and 13L. Recent studies have pointed out that the forced swim test is related to a much broader range of neurobehavioral conditions than just depression^28^. In the forced swim test, rodents are faced with an inescapable aversive situation. Active coping strategies include climbing and swimming, while the passive strategy is floating^28^. Therefore, a longer immobility time indicates that the injected mice are depressed or that they apply different stress-coping strategies in the water. Intriguingly, application of an atypical antidepressant agomelatine attenuated anxiety and depressive-like responses in a rat model of the early stage of Alzheimer’s disease, which was accompanied by neuroprotection in the piriform cortex^46^. Therefore, vacuolar degeneration in the piriform cortex in 13S- and 13L-injected mice might be involved in the behavioral changes. However, we cannot exclude a possibility that undetectable changes in other brain regions are also responsible to the behavioral changes. The behavioral tests were performed already 1 day after the injection. We consider it, however, unlikely that postoperative damage is solely responsible for the altered behavior as significant differences between the 13S and scrambled serine peptides were detected at this time point.

The vacuolar degeneration was also observed in the motor cortex, striatum and hippocampus of 13S-treated mice. These brain regions control and modulate motor activities. Nevertheless, spontaneous locomotion was not changed in mice given 13S and 13L, as evidenced by open field test. Thus, neurons in the piriform cortex might be functionally damaged more than the other brain regions. Future studies using electron microscopy are hence required to specify the ultrastructural changes that are responsible for emotion.

## Methods

### Peptides

The purity of the synthesized TAMRA-labeled peptide with 13 serine repeats (13S) and scrambled serine (GL Biochem, Shanghai, China) were more than 95%, whereas, that of the TAMRA-labeled peptide with 13 leucine repeats (13L)(GL Biochem, Shanghai, China) was 75%, the best purity to achieve. The sequences were 5-TAMRA-KKWSSSSSSSSSSSSSKK-NH_2_, 5-TAMRA-KKWLLLLLLLLLLLLLKK-NH_2_ and 5-TAMRA-SSSKSSKSKSSSWSSSKS -NH_2_ for 13S, 13L and scrambled serine, respectively. The flanking sequences of 13S and 13L were the same as those described in a previous study^22^. The peptide powder was first dissolved in a 1:1 mixture of trifluoroacetic acid and hexafluoroisopropanol.

### Cell culture

PC12 and KT-5 cells were purchased from RIKEN BRC. For in vitro experiments, 13S peptide and scrambled serine peptide (1 mg/ml in water) was incubated for 48 h at 37 °C with shaking before addition to the culture medium. Then, the aggregated peptides were added to culture medium of cells to be at a final concentration of 10 µg/ml. For 13L, the stock solution of 13L peptide was directly applied in the culture medium to be at a final concentration of 10 µg/ml.

For PC12 cell culture, the micro cover glass was coated with laminin (30 µg/ml). Then, PC12 cells were plated in 24 well plates with the micro cover glass inside the setup. The cells were cultured in DMEM containing 10% FBS and antibiotics essentially as described^23^. The next day, the cells were induced to differentiate by the addition of NGF (50 ng/ml) in DMEM containing 1% FBS and 0.25% BSA and were cultured for 5 days. Then, peptides were added to the culture medium and were further cultured for 1 day and 4 days without NGF for cell viability and morphological analyses, respectively. Cell viability was also tested using undifferentiated PC12 cells.

For KT-5 cell culture, the cells were cultured in Ham's F12 medium with 4 mM HEPES, 10% FBS and antibiotics in 24-well plates for 4 days after the addition of peptides. The cells were then subjected to cell viability and morphological analyses.

Viability of cells in 96-well plates was quantitatively measured using the Cell Counting Kit-8 (Dojindo, Kumamoto, Japan) according to the manufacturer’s instructions. Absorbance at 450 nm was measured using a microplate reader and the values relative to those of TAMRA-treated cells were expressed.

### Quantification of aggregates and cultured cells

The total length of neurites and number of branch points of the neurites of PC12 cells were measured using Image J software as described previously^24^. For quantification of KT-5 cells, the total area of cells including the cell body and average length of each process per cell were also measured using Image J software. Since length less than 1.0 µm was difficult to detect, we set the shortest detection limit at 1.0 µm. Area of aggregates were also measured using Image J software.

### Transmission electron microscopy

Fixation of PC12 and KT-5 cells was performed using 2% paraformaldehyde (PFA) and 2% glutaraldehyde (GA) in 0.1 M phosphate buffer (PB), pH 7.4 at 4 °C for 30 min followed by treatment with 2% GA in 0.1 M PB at 4 °C overnight. The next day, the cells were postfixed with 2% osmium tetroxide in 0.1 M PB for 1 h at 4 °C. After dehydration with graded alcohol, the cells were transferred to a resin (Quetol-812; Nisshin EM Co., Tokyo, Japan) and polymerized for 48 h at 60 °C. Ultra-thin sections of the polymerized resins (70 nm in thickness) were prepared using an ultramicrotome (Ultracut UCT; Leica, Vienna, Austria) and a diamond knife. The sections on copper grids were stained with 2% uranyl acetate for 15 min at room temperature followed by staining with lead stain solution (Sigma) for 3 min at room temperature. A transmission electron microscope (JEM-1400Plus, JEOL Ltd., Tokyo, Japan) was set at an acceleration voltage of 100 kV. Digital images with 3296 × 2472 pixels were taken from a CCD camera (EM-14830RUBY2, JEOL Ltd., Tokyo, Japan).

### Mice

The animal study was performed in compliance with the ARRIVE guidelines. Animal experiments were approved by the Animal Resource Committees of Gunma University. We followed the NIH guidelines for the treatment of the animals. The mice were kept in specific pathogen-free conditions in a room where the temperature and light/dark cycle were set at 23 °C and 12 h, respectively. The number of mice used was minimum required to obtain reliable data and made every effort to minimize the suffering of mice. Adult ICR mice (older than 2 months old) were anesthetized with isoflurane and fixed by the ear bars of the stereotaxic instrument. Then, the mice underwent injection of peptides (5 µl) into right lateral ventricle (anteroposterior + 0.2 mm, lateral + 0.8 mm to bregma, and dorsoventral -2.5 mm below dura with the skull leveled between lambda and bregma) essentially as described previously^47^. 13S peptide and scrambled serine peptide (1 mg/ml) were induced to aggregate by incubation for 48 h at 37 °C with shaking and then diluted to 100 µg/ml with PBS for the injection. For 13L, the stock solution of 13L peptide was directly diluted to 100 µg/ml with PBS for the injection. Behavioral tests were performed the following day.

### Imaging

Fluorescent staining was carried out essentially as described previously^23^. Coronal brain sections 20 µm in thickness were prepared using a cryostat and these were fixed with 4% PFA for 10 min at room temperature for fluorescent staining. The brain sections were incubated with DAPI and Phalloidin-iFluor 488 conjugate (Cayman Chemical, Ann Arbor, MI). The fluorescent images were captured using LSM 880 confocal microscope (Zeiss, Oberkochen, Germany). For HE and Nissl staining, mice were transcardially infused with 4% PFA and the brains were postfixed with the same fixative solution overnight. After dehydration with 30% sucrose, the coronal brain sections 20 µm in thickness were prepared using cryostat. For Nissl staining, the sections were stained with 0.1% cresyl violet solution for 20 min at 60 °C. The visible images were obtained using a BZ-9000 microscope (Keyence, Osaka, Japan).

### Behavioral tests

Spontaneous locomotion of mice was automatically measured for 10 min using an apparatus (50 cm × 50 cm × 50 cm, O’HARA & CO., LTD., Tokyo, Japan). The floor of the open field was covered with black paper to enable the automatic detection of white mice by the camera system. The mouse was first placed in the center of the square open field, and then allowed to walk. The locomotion parameters measured in the system were total walking distance, duration of movement for 10 min, total number of movements, average speed of locomotion for 10 min, speed during movement, walking distance per movement and duration per movement. The percentage of time the mice stayed in the center area was also recorded during this experiment.

The elevated plus maze consisted of two open and two closed arms (each 25 cm long and 5.5 cm wide) and a central area (5.5 cm × 5.5 cm). The closed arms had transparent walls that were 14.5 cm in height. The maze was placed at 55 cm above the ground. Mice were initially placed in the center area facing an open arm and were allowed to enter the arms. The total time spent in the open arms was measured for 10 min. In addition, the percentage of the number of entries into the open arms out of the number of entries into all four arms was also calculated. Entry into the arms was considered when the hind limbs of the mice were placed in the arms.

The forced swim test was performed as described previously^48^. The depth of water in the circular tank (11.3 cm in diameter and 21.5 cm in height) was set at 15 cm. Mice were first acclimated in the water at room temperature for 2 min. After the acclimation period, the total immobility time was measured for 6 min. The three tests above were carried out on the same day in the following order; open field test, elevated plus maze test and then forced swim test.

### Statistical analysis

The values are expressed as the mean and the error bars represent SE in the graphs. Statistical significance was studied using ANOVA followed by Tukey or Tukey–Kramer posthoc tests. *p* values less than 0.05 were considered as statistically significant.

## Supplementary Information

Supplementary Figure S1.

## Data Availability

All data generated or analyzed during this study are included in this published article.

## Abbreviations

polyL: Polyleucine
polyQ: Polyglutamine
polyS: Polyserine
RAN: Repeat-associated non-AUG
