# Supplementary figures and images for: Exogenous polyserine and polyleucine are toxic to recipient cells
Source: Sci Rep. 2022 Jan 31;12:1685. doi: 10.1038/s41598-022-05720-y (PMC8803884; doi:10.1038/s41598-022-05720-y)

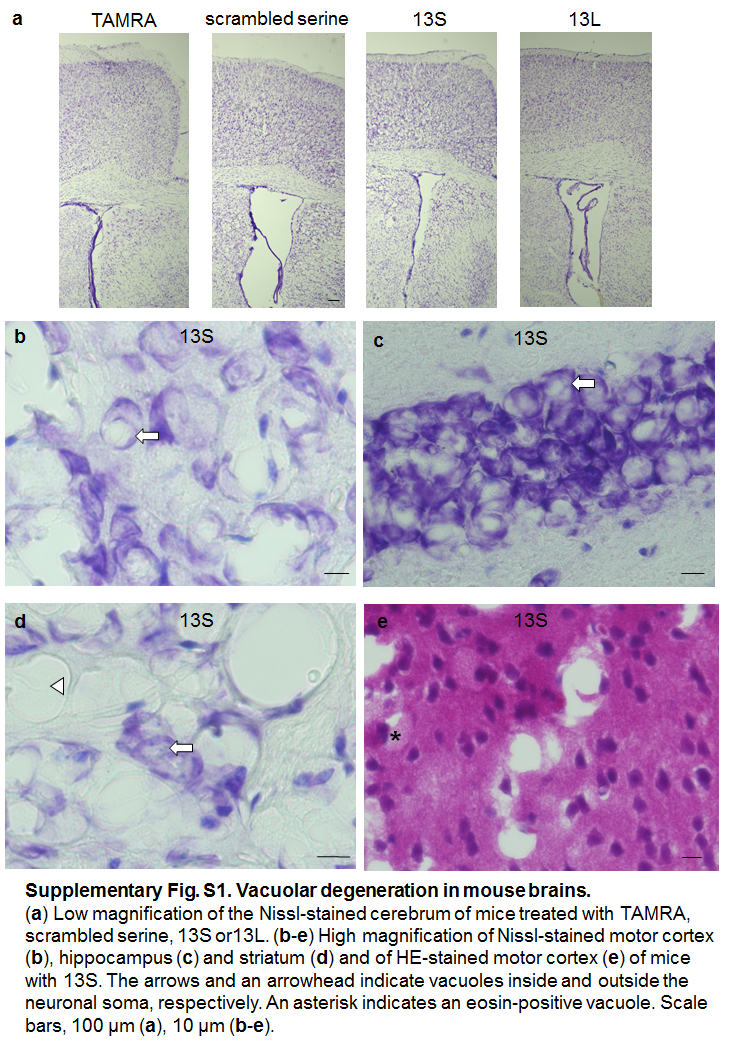

Supplement: Supplementary file 1 — Supplementary Figure S1. [file 41598_2022_5720_MOESM1_ESM.tif]
